# Supplementary material for: Conditional chemoconnectomics (cCCTomics) as a strategy for efficient and conditional targeting of chemical transmission
Source: eLife. 2024 Apr 30;12:RP91927. doi: 10.7554/eLife.91927 (PMC11060718; doi:10.7554/eLife.91927)
Supplement: Figure 3—source data 3. [file elife-91927-fig3-data3.zip › Figure 3 source data3 part 1.zip!/Figure 3-Source data 3-part1/1082-9-CRISPResso_Report_zod2ewbs.zip!/CRISPResso_on_1082_12/3b.Insertion_deletion_substitutions_size_hist.pdf]

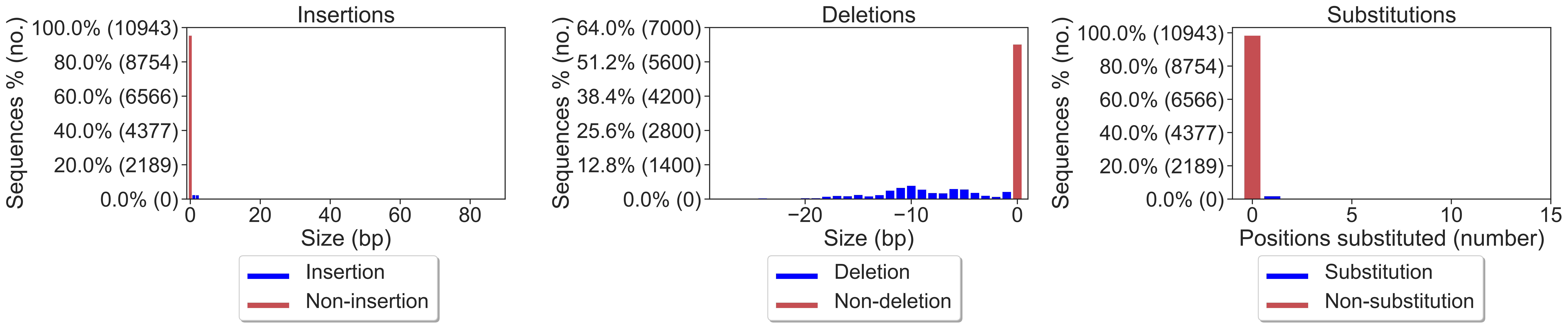
Insertions
Sequences % (no.)
100.0% (10943)
80.0% (8754)
60.0% (6566)
40.0% (4377)
20.0% (2189)
0.0% (0)
0
20
40
60
80
Size (bp)
Insertion
Non-insertion
Deletions
Sequences % (no.)
64.0% (7000)
51.2% (5600)
38.4% (4200)
25.6% (2800)
12.8% (1400)
0.0% (0)
−20
−10
0
Size (bp)
Deletion
Non-deletion
Substitutions
Sequences % (no.)
100.0% (10943)
80.0% (8754)
60.0% (6566)
40.0% (4377)
20.0% (2189)
0.0% (0)
0
5
10
15
Positions substituted (number)
Substitution
Non-substitution
